# Towards proton therapy guidelines for radiation therapists and dosimetrists: A scoping review^☆^

**DOI:** 10.1016/j.tipsro.2025.100322

**Published:** 2025-07-13

**Authors:** E. van Weerd, J.J. Jacobs, A.M. Moerman, C. Xavier, I.T. Kuijper, N. de Nie, H. Bijwaard, M.S. Hoogeman

**Affiliations:** a Holland Proton Therapy Centre, Huismansingel 4, 2629 JH Delft, the Netherlands; b Inholland University of Applied Sciences, Bijdorplaan 15, 2015 CE Haarlem, the Netherlands; c National Institute for Public Health and the Environment (RIVM), Antonie Van Leeuwenhoeklaan 9, 3721 MA Bilthoven, the Netherlands; d Department of Radiotherapy, Erasmus MC Cancer Institute, University Medical Center Rotterdam, Dr. Molewaterplein 40, 3015 GD Rotterdam, the Netherlands

**Keywords:** Guideline, Consensus, Best practice, Radiation therapist, Dosimetrist, Proton therapy

## Abstract

With the increasing number of proton therapy centers worldwide, particularly in Europe, proton therapy is becoming a more established treatment option. However, education and guidelines, specifically tailored to radiation therapists (RTTs) and dosimetrists, are lacking. Through the “Towards a Sustainable RTT Network” (TaSeRnet) project, efforts are underway to harmonize proton therapy practices among RTTs and dosimetrists across Europe. This scoping review aims to identify and summarize existing guidelines relevant to RTTs and dosimetrists working in proton therapy, providing a necessary first step toward the future development of specific guidelines and education for these professions. Ten articles were identified that include guidelines covering certain aspects of proton therapy workflows in several clinical indications. However, significant gaps remain regarding the specific tasks performed by RTTs and dosimetrists. In particular, no guidelines were found addressing treatment execution, a workflow typically performed by RTTs. Moreover, the limited involvement of RTTs and dosimetrists in the development of existing guidelines may result in the exclusion of essential practical knowledge and expertise. As they play a critical role in the daily delivery of proton therapy, their input is vital to ensure comprehensive and applicable guidelines. In conclusion, this review underscores the need for developing guidelines specifically for RTTs and dosimetrists in collaboration with the broader multidisciplinary team. Developing such guidelines will support the standardization of clinical practice and contribute to improved quality of care.

## Highlights

• RTT-specific workflow steps are missing in existing proton therapy guidelines.
• Guidelines for RTTs in proton therapy are lacking and need to be developed.
• Multidisciplinary collaboration is crucial for RTT-focused guideline development.
• RTT-focused guidelines are essential for improving the quality of proton therapy.

## Introduction

The number of proton therapy centers (PTCs) is growing rapidly, particularly in Europe, establishing proton therapy (PT) as a well-recognized treatment option [1]. Despite this growth, education and guidelines, specifically for radiation therapists (RTTs^1^), have lagged behind [2]. Currently, education is often developed and provided exclusively within individual PTCs with only limited national programs available. Furthermore, guidelines are mainly created by radiation oncologists (ROs) and medical physicists (MPs) and the involvement of RTTs in the development of guidelines remains limited [3]. This could lead to the creation of guidelines that may not fully address RTT-specific needs. In a rapidly advancing technological environment, standardized education and comprehensive guidelines are essential to ensure consistent practices and improve the quality of care [4]_._ Without these resources, practice variability may increase, which could potentially have a negative impact on clinical outcomes and patient experience [5,6,7].

To address these challenges, the “Towards a Sustainable RTT network” (TaSeRnet) project was initiated with funding from the European Union under the Erasmus+ Program. RTTs from various PTCs are collaborating to identify best practices and establish guidelines, aiming to harmonize PT planning and delivery across Europe [8]. The Delphi methodology will be used to achieve consensus on these best practices. To provide a foundation for the Delphi methodology, it is essential to assess which best practices and guidelines have already been established and agreed upon. Therefore, this review aims to identify relevant guidelines and recommendations from the past decade specifically related to the tasks performed by RTTs in PT. Although the TaSeRnet project focuses on harmonizing PT practices across Europe, this review aimed at finding best practices and guidelines worldwide.

## Material and methods

To identify guidelines^2^ related to the work of RTTs, workflow steps were categorized as defined in the TaSeRnet project framework, which includes patient simulation, image processing, treatment planning and evaluation, quality assurance, setup and verification, treatment execution and evaluation during treatment course [8].

### Literature search

This scoping review was performed using the steps as described by Mak et al. [9]. The literature search was conducted on April 5th 2024, using PubMed and Cochrane databases. A search strategy was developed in consultation with a librarian. Google Scholar and article references were used to ensure data saturation. Table 1 provides an overview of the search terms used, which were derived from an initial search on Google Scholar. The search focused on PT, best practices and pencil beam scanning (PBS), which is the predominant technique in PT [1]. Just before submitting the article, the literature search was repeated on November 25th 2024, using the same methodology as the first search.

**Table 1:** Overview of the used search terms combined using OR within columns and further combined using AND across columns to construct the search strategy.

*Proton therapy*	*AND*	*Best practice*	*AND*	*Technique*
Proton radiation therapy		Consensus		Pencil beam scanning
Proton therapy		Best practice*		Spot scanning
Proton beam therapy		Harmonization		Beam scanning
Particle beam therapy		Guideline*		Pencil beam scanned
Particle therapy		Practice pattern*		Scanning system
Proton beam radiation therapy				IMPT
Proton treatment				Intensity modulated proton therapy
				Active delivery

*Is used to search for singular and plural.

### Selection criteria

Articles were selected based on the following inclusion criteria:

- Describing best practices, recommendations, guidelines, or consensus.
- Pencil beam scanning.
- English language.
- Published less than ten years ago.

Articles were excluded if they concerned:

- Practices not performed by RTTs.
- Passive scattering PT.
- Ocular proton therapy
- No clinical setting (e.g., phantom study).
- Other particles than protons.
- No full text available.

It remains challenging to define the specific tasks and responsibilities of RTTs. In 2014, the European Society for Radiotherapy and Oncology (ESTRO) developed a benchmarking document outlining the knowledge, skills, and competencies of RTTs [10]. However, these descriptions are general, and variations in tasks and responsibilities may still exist across countries and institutions. Therefore, the search strategy was not specifically designed to identify guidelines for the work domains of RTTs, but rather focused on guidelines established for the entire field of PT. The authors then extracted results applicable to the tasks and responsibilities of RTTs.

Two review authors (E. van Weerd and J.J. Jacobs), both RTTs with advanced practice roles and extensive experience in PT preparation and delivery, independently reviewed titles, and abstracts to identify relevant articles. Disagreements were resolved through discussion. If consensus could not be reached, a third RTT (I.T. Kuijper), expert in photon radiotherapy and lecturer in PT for the Dutch master’s program, was available to make the final decision.

### Data extraction

Data was independently extracted from the selected articles by the two review authors using an extraction table. The extracted data included author, year of publication, journal, population, workflow steps, methods, results and limitations.

### Quality score

All articles were evaluated by the two review authors using the AGREE II instrument (Appraisal of Guidelines for Research and Evaluation), which assesses the quality of clinical guideline development across six domains [11]. Each domain was scored from 0% to 100% based on the reviewers’ evaluations. The purpose of this assessment was not to exclude articles from this review based on the quality score, but rather to assess the quality of the guideline itself.

## Results

**Fig. 1:**
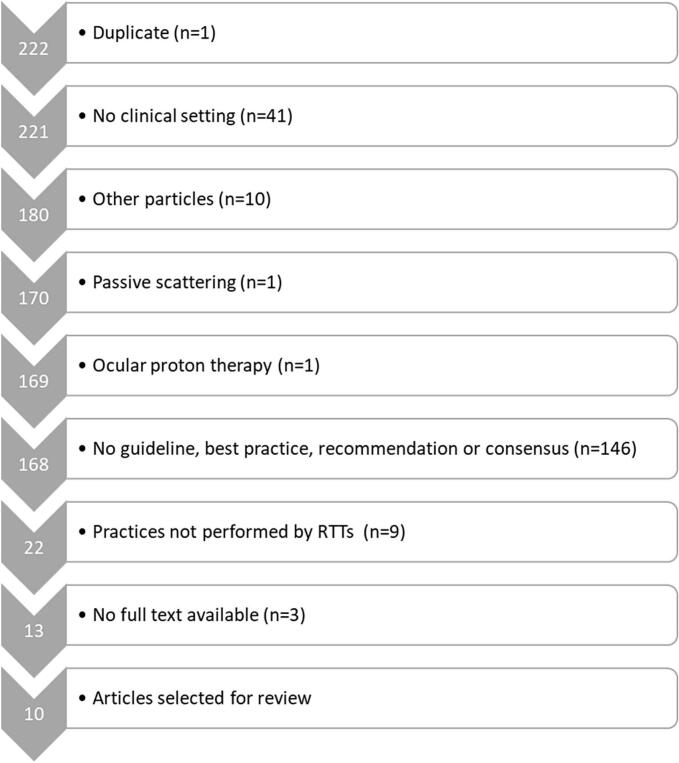
Flowchart illustrating the literature selection process. The left column displays the number of articles included at each step. The exclusion criteria and corresponding number of excluded articles (n) are shown on the right.

A total of 222 articles were identified through the literature search. After applying the in- and exclusion criteria, 10 articles, published between 2017 and 2024, were selected for data extraction (Fig. 1).

The 10 selected articles addressed various indications, as summarized in Table 2. Eight articles developed guidelines or recommendations, one article defined the minimum requirements for the implementation of IGPT procedures based on a Delphi consensus, while one article summarized existing literature to provide guidance for clinical practice and future research. The guidelines were primarily based on literature reviews, two articles also included clinical findings and experience into the development process and four articles incorporated expert consensus.

**Table 2:** Overview of included articles, detailing authors, journal, target population, the basis of the guidelines and the aim of each article.

*Author (year)*	*Journal*	*Population*	*Based on*	*Aim*
Arjomandy (2019)	Medical Physics	General	Literature and common practice	Guidelines and recommendations
Bryant (2021)	International journal of particle therapy	Prostate cancer	Literature	Summary of results literature
Chang (2017)	Radiation oncology, biology, physics	Thoracic malignancies	Physics and clinical findings	Guidelines and recommendations
Chhabra (2024)	Radiation oncology, biology, physics	Spinal Tumors	Literature	Recommendations
Choi (2024)	International Journal of Particle Therapy	Lower gastrointestinal	Literature and consensus	Guidelines and recommendations
Chuong (2021)	Frontiers in Oncology	Esophageal cancer	Literature	Guidelines
Dabaja (2018)	Blood	Mediastinal lymphomas	Literature review and consensus	Guidelines
Mutter (2021)	Radiation oncology, biology, physics	Breast cancer	Literature review, clinical experience, and consensus	Guidelines and recommendations
Trnková (2024)	Physica Media	Cranio-spinal irradiation	Delphi consensus	Minimum requirements
Zeng (2020)	Elsevier	Mesothelioma	Literature	Guidelines

Table 3 outlines the workflow steps covered by each article. All but one article discusses several workflow steps, except for the study by Arjomandy et al., which focuses on QA. No guidelines addressed treatment execution. The results of each workflow step are described in sections 3.2 through 3.7.

**Table 3:** Visualization of the included articles, showing the workflow steps (left column) and the indications (top row), with each article mapped to the corresponding workflow step and indication it addresses. CSI = cranio-spinal irradiation, LGI = lower gastrointestinal.

	*Thorax*	*Prostate*	*Breast*	*CSI*	*LGI*	*Spine*	*General*
Patientsimulation	Chang et al.	Bryant et al.	Mutter et al.	Trnková et al.	Choi et al.	Chhabra et al.	
	Chuong et al.						
	Dabaja et al.						
	Zeng et al.						
Imageprocessing	Chang et al.		Mutter et al.	Trnková et al.	Choi et al.	Chhabra et al.	
	Chuong et al.						
Treatmentplanning andevaluation	Chang et al.		Mutter et al.	Trnková et al.	Choi et al.	Chhabra et al.	
	Chuong et al.						
	Dabaja et al.						
	Zeng et al.						
Qualityassurance	Chang et al.						Arjomandy et al.
	Chuong et al.						
Setup andverification	Chang et al.	Bryant et al.	Mutter et al.	Trnková et al.	Choi et al.	Chhabra et al.	
	Chuong et al.						
	Dabaja et al.						
	Zeng et al.						
Treatment execution							
Evaluationduringtreatmentcourse	Chang et al.	Bryant et al.	Mutter et al.	Trnková et al.	Choi et al.	Chhabra et al.	
	Chuong et al.						
	Dabaja et al.						
	Zeng et al.						

### Quality score

Table 4 shows the AGREE II quality scores. Six articles scored good to excellent for scope and purpose. Four articles with lower scores lacked detailed description of the target population and the absence of a clearly stated purpose for the guideline. Stakeholder involvement scored poor to fair in six articles due to insufficient description of the working group composition, the failure to indicate whether the guidelines were reviewed by the target population, and the lack of clarity regarding the intended users of the guideline. Seven articles scored poorly on the rigor of development. Although references suggest that a literature search was conducted, the methodology for this search was not provided. Clarity of presentation was rated good to very good in nine articles, but applicability scored poorly to fair across all. No articles provided tools for guideline application or addressed implementation barriers or costs. One article did not mention financial or other disclosures. Two articles explicitly reported having no disclosures. The remaining articles reported disclosures but did not specify their influence on guideline development; in three of these, only financial or non-financial disclosures were mentioned.

**Table 4:** Quality score (%) according to AGREE II per domain and article.

### Patient simulation

#### Scanning protocols

##### Scanning protocols

Consensus was reached on using single- or dual-energy computed tomography (CT) for cranio-spinal irradiation (CSI) treatment planning (TP) and implementing dedicated pediatric protocols for children [12].

##### Contrast agent

A non-contrast CT scan is used for TP [12,13,14], with an optional contrast-enhanced CT to aid delineation [11,12]. Contrast should be administered after the planning CT to avoid its impact on Hounsfield Unit (HU) numbers used for dose calculation [14]. For esophageal patients, oral contrast is avoided to prevent stomach distention [14].

##### Scan length

The scan volume should encompass the entire body surface, positioning devices and organs at risk (OARs) which are important for dose evaluation [14,15]. Scan length and slice thickness for several indications are summarized in Table 5.

**Table 5:** Recommended scan length and slice thickness for several indications (with subgroups in italic).

*Author*	*Population*	*Scan length*	*Slice thickness (mm)*
Chhabra et al.	Spinal tumors *Cranial region*	Target area *NR*	≤ 3 < 3
Choi et al.	Lower gastrointestinal	From mid-femur to L2-L3 or 5 cm above nodal disease	≤ 3
Chuong et al.	Esophagus *Upper mediastinum + cervical lymph nodes*	Include entire lungs and kidney Include full neck to skull base	≤ 3
Zeng et al.	Mesothelioma	*NR*	≤ 3

NR = not reported.

##### Foreign materials

Metal artifact reduction algorithms are recommended to reduce artifacts and improve dose calculation accuracy [13,16]. These algorithms should be carefully evaluated [17]. Dual-energy CT or higher energy CT scans may also be considered [13].

##### Patient instructions

Recommendations on patient instructions are summarized in Table 6. In LGI treatment, vaginal dilators can be considered for sparing the anterior vaginal wall and urethra. In such cases, an additional scan without a vaginal dilator may be performed to evaluate robustness if the dilator is no longer tolerated during treatment. Similarly, an empty bladder scan may be performed alongside the full bladder CT scan to assess robustness if bladder filling is not achieved during treatment [17].

**Table 6:** Patient instruction and supportive tools for several indications.

*Author*	*Population*	*Patient instruction*	*Supportive tools*
Bryant et al.	Prostate	Full bladder, empty rectum	Rectal saline, rectal balloons, perirectal spacers
Choi et al.	Lower gastrointestinal	Full bladder (empty bladder upon arrival and drink 475 ml of water around 30–60 min before appointment), empty rectum	Low-gas diet, anti-foaming agent, anti-gas agent, vaginal dilator, nonmetallic radiopaque markers to mark anal verge
Chuong et al.	Esophagus	Empty stomach	*NR*

NR = not reported.

##### Motion management

A four-dimensional (4D) CT scan is recommended to evaluate motion [14,15,18,19]. Establishing acceptable tumor motion criteria is advised to determine the appropriate motion strategy [18]. Patients can be treated in free breathing if target motion is under 5 mm [15,19]. Otherwise, motion mitigation strategies are advised [14,15,19]. When using a breath hold (BH) technique or mechanical ventilation, the 4D-CT should reflect treatment conditions, requiring multiple BH scans to ensure reproducibility and define an internal target volume (ITV) [14,19]. In mediastinal lymphomas, deep inspiration BH (DIBH) can reduce mediastinal motion and reduce lung and heart dose [19]. For breast cancer patients, DIBH may only benefit selected patients [16].

#### Optimal patient setup and immobilization

An optimal patient setup minimizes anatomical and setup variations, ensures reproducibility, and is well-tolerated [13,15]-[17,19]. It also provides access for appropriate beam angles [13]. Table 7 summarizes setup recommendations for specific indications. For breast cancer patients with limited arm mobility, an arms-down position can be considered [16]. Chuong et al. recommend a pre-simulation multidisciplinary meeting to discuss immobilization and motion mitigation techniques. In esophageal patient setup, attention should be given to gastric tumor extension, as the left arm may be in the beam path [14]. For LGI and sacral tumors, prone positioning moves the bowel away from high-dose areas and is preferred when treating through the table is limited, or additional angles are needed for skin sparing [13,17]. However, supine position, is generally more comfortable, reproducible, and reduces respiratory motion [13,17]. In LGI treatment, care should be taken to position the genitalia out of the field [17].

**Table 7:** Patient orientation, arm position and immobilization devices for several indications (with subgroups in italic).

*Author*	*Population*	*Patient orientation*	*Arm position*	*Immobilization devices*
Choi et al.	LGI *Inguinal*	Supine or prone Supine	*NR* *NR*	Thermoplastic mold for prone position
Chuong et al.	Esophagus *Cervical*	Supine Supine	Up (preferred) Down (alternative) Down	Full body immobilization device or immobilization of the hips Thermoplastic mask
Chhabra et al.	Spinal tumors *Sacral* *Thoracic* *Cervical*	Supine Prone Supine Supine	*NR* *NR* *NR* *NR*	Belly board Custom pillow Thermoplastic mask
Mutter et al.	Breast	Prone or supine	Up or down	*NR*
Trnková et al.	CSI	Supine or prone	*NR*	Thermoplastic mask and knee support
Zeng et al.	Mesothelioma	Supine	Up	*NR*

NR = not reported.

### Image processing

#### Registration of images

Registration of diagnostic imaging to the planning CT may assist target and OAR delineation [12]-[14,16,17]. The optimal registration method is either region-specific rigid registration [12] or deformable registration [17]. A diagnostic magnetic resonance imaging (MRI), positron emission tomography (PET)-CT or a diagnostic CT scan may be registered for esophageal, breast, CSI, LGI and spinal tumors [12]-[14,16,17]. Additionally, an adequately registered T1- and T2-weighted MRI scan or CT myelogram in treatment planning position can help to accurately delineate the target and spinal cord in spinal tumors. In case of re-irradiation, importing previously irradiated treatment plan helps identify dose hot spots and avoid high cumulative dose in critical OARs [13].

#### Contouring of organs at risk

Chuong et al. recommend performing the delineation of OARs in esophageal patients on the non-contrast 4D-CT dataset. Typically, the average-CT scan is used, but alternatively the end exhale phase can be utilized [14]. In spinal tumors the center of the spinal cord is delineated as a 2 mm tubular structure at the center of the spinal cord contour [13].

### Treatment planning and evaluation

#### Generation of plans

##### Beam setup

When selecting beam angles, general considerations should be taken into account:

- Shortest and homogeneous beam path possible [18].
- Smallest value of the maximum temporal water equivalent thickness (WET) change in the beam path [14,18].
- Patient anatomy, geometry and target location [18].
- To not let the beam stop into critical structures, especially structures effected by motion interplay or anatomic changes [18,19].

##### Beam setup thorax

For thoracic malignancies motion analysis is recommended to select beam angles parallel to the primary direction of target motion. In case of reirradiation, it is advised to limit the dose to previously irradiated tissue and choose beam angles that pass through nonfunctional or fibrotic lung tissue [18].

##### Beam setup esophagus

For esophagus cancer, posterior beam angles are most robust against motion. To spare OARs lateral to the target, superior-inferior posterior oblique beams with a 270° couch angle can be used, though this increases spinal cord dose. For cervical and proximal located targets, an anterior beam may be used to reduce lung dose. In case of split target volumes, dose homogeneity at the junction should be evaluated in the presence of setup errors and respiratory motion [14].

##### Beam setup mediastinal lymphoma

Beam setup for mediastinal lymphoma depends on the target location. Posterior fields are preferred for posterior targets, and anterior fields for anterior targets, avoiding both directions to prevent unnecessary irradiation of the heart and breasts. For complex target volumes, involving posterior and anterior located targets, a combination of field directions can be used to spare heart and lungs. In cases with anterior upper mediastinal and lower neck targets, one beam with repainting or two anterior fields may be used. For axillary involvement, posterior fields are recommended. The target can also be divided into two or more parts with different beam directions, in which case dose homogeneity at the junction should be evaluated. Multiple beams are generally recommended to improve dose conformality and reduce uncertainties [19].

##### Beam setup breast

For the beam setup in breast cancer patients, it is recommended to use multiple frontal fields, to minimize dose to healthy tissue and achieve uniform skin sparing. Two to three beams are used to avoid hotpots in linear energy transfer (LET) from beams ranging in the ribs, heart and lungs [16].

##### Beam setup mesothelioma

In the TP of mesothelioma two to four fields are used. The beams range from anterior to posterior around the ipsilateral chest [15].

##### Beam setup LGI

For anorectal targets, left and right posterior oblique angles are preferred, as they avoid regions with variations in bowel gas and minimize dose to the bowel and bone marrow. However, these angles can be affected by anatomical and setup variations. Lateral beams are also robust but do not spare the bone marrow. For inguinal nodes, anterior-posterior or left/right anterior oblique beams may be used. In the case of hip implants, beams that traverse or pass near the implant should be avoided. Split target-based optimization can be used to improve bowel and bladder sparing [17].

##### Beam setup spinal tumors

For mobile spine and sacral tumors, two or three posterior beams are used to minimize beam path length, reduce OARs dose, and avoid changing anatomy such as the bowel or lungs. For cervical spine tumors, an additional anterior-oblique beam may be used, provided that variations in chin and shoulder positioning are accounted for. Because of the worse lateral penumbra of proton beams, compared to photon beams, it may be beneficial to either use a mixed photon-PT technique or apertures to reduce the lateral penumbra [13].

##### Objective use

In mediastinal lymphoma it is important to keep the dose to the breasts as low as possible, especially in young patients [19]. In breast TP a constraint for the esophagus and brachial plexus may be used to mitigate the risk of radiobiological effect (RBE) heterogeneity [16]. For mesothelioma TP it is recommended to minimize hot spots and dose to the skin [15]. For spinal tumors, spinal cord, cauda equina and sacral nerve roots are of critical importance and may be robustly optimized [13].

##### LET/RBE uncertainties

Chhabra et al recommend avoiding beam angles that intersect the spinal cord and other critical organs [13]. Choi et al. and Chhabra et al recommend considering biological uncertainty during optimization, if this is available, to remove high LET peaks away from the OARs [13,17]. If RBE- or LET-based TP is unavailable, Dabaja et al. proposes strategies to mitigate potential increase in RBE by [19]:

- reducing physical dose to OARs at the distal end of the beam.
- using multiple fields.
- range feathering.
- irradiating past sensitive structures.
- allowing respiratory and cardiac motion to feather out the dose for moving OARs.

##### SFO/MFO techniques

Single field optimization (SFO) reduces motion uncertainty and might therefore be considered for thoracic indications treated with PBS, especially when 4D-optimization and 4D-evaluation are unavailable or when motion amplitudes range between 5–10 mm. For patients with less motion or when motion mitigation techniques are applied, and in breast, LGI, mesothelioma and spinal TP, an MFO technique is recommended to improve target coverage and OAR sparing [13,15]-[18]. When MFO is used, attention must be paid to the dose gradients in the treatment field to ensure homogeneity in the presence of setup errors and motion [14,17]. For spinal tumors two approaches are suggested. In the first approach multiple treatment plans with varying robustness or modulation levels are delivered to ensure plan robustness while maintaining adequate sparing of OARs. In the second approach, if the plan is sufficiently robust, MFO may be considered throughout the entire treatment course [13].

##### Foreign materials

In the case of foreign materials, it is recommended to accurately determine their composition, which can be assessed through a review of the operative report [13,16]. For small or thin metal implants with complex shapes, accurate delineation may be challenging and overriding HU values may exaggerate the dosimetric impact [13]. When accurate HU values are unavailable, the material should be delineated and avoided. Chhabra et al. recommend expanding blocking structures by several millimeters to account for setup uncertainties. If avoiding foreign material is not feasible, increasing the number of beam angles can reduce uncertainties, while large hinge angles help minimize overlap behind the material. For certain patients, alternating delivery of multiple treatment plans with slightly varying beam angles, may be beneficial. If the TP system is commissioned for accurate stopping power prediction in the presence of foreign materials, shooting through the material may be a viable option. However, care must be taken with objects that may be damaged by proton irradiation or high doses [13]. Using a Monte Carlo dose algorithm and high-resolution dose grid enhances dose calculation precision. A density override is suggested as a mitigation strategy for metal implants and artifacts, although range uncertainties should be carefully evaluated [12,13,17]. Beam angles that avoid artifacts are preferred, if not feasible, angles perpendicular to artifacts can be used instead [13].

##### Plan parameters

Zeng et al. recommends using a Monte Carlo dose calculation algorithm to ensure accurate dose calculation [15].

##### Motion management

When treating thoracic malignancies in free breathing, the average CT can be used for TP with an internal gross target volume (iGTV) approach with a density override using an average HU inside the solid GTV [15,18]. For esophageal cancer it is recommended to use the same motion management strategy as for lung cancers [14]. In free breathing treatment it is advised to use appropriate motion-robust planning methods [14]. If the target is affected by motion, it is recommended to use an ITV [19]. Techniques to mitigate the interplay effect include repainting, increase spot size, gating or use of an optimized delivery sequence, including scanning direction and breath sampling [14,18,19]. It is important to recognize that SFO with multiple fields or administering fractionated treatment can also effectively mitigate the interplay effect [18]. According to Chang et al. repainting should be mandated in hypo fractionated treatments [18]. If BH or gating is used, and the residual target motion is greater than the established threshold value, it is advised to validate treatment plans on either an additional BH-CT or another phase of the 4D-CT [18].

#### Robust against uncertainties

Uncertainties should be accounted for by applying margins to the clinical target volume (CTV) for setup and range uncertainties [13,14,16,17,19]. Robust optimization and evaluation, in which also anatomical variations can be simulated, can minimize the need for replanning [16,17,19]. A planning OAR volume may also be used, depending on the institutional preferences [13]. In the robustness evaluation, planning criteria should be met for targets and OARs [14,17]. Although, some deviation from nominal constraints may be allowed [13].

For thoracic indications, specific guidelines have been developed on robust optimization and robust evaluation against motion. Chang et al. recommend deformable registration of the extreme breathing phases to assess motion and evaluate the potential effects of intrafraction motion on the treatment plan. This evaluation may guide decisions on motion mitigation strategies. 4D accumulated dose (4DD) and 4D dynamic accumulated dose (4DDD) can be used to assess the interplay effect and the effectiveness of motion mitigation strategies [15,18]. If the criteria for target coverage cannot be met, techniques to mitigate the interplay effect may be applied.

Robust optimization minimizes the impact of organ motion, and 4D robust optimization further improves robustness, especially against large intrafraction motion and density changes along the beam path [14,15,18]. The latter is also recommended for short fractionation schemes [18].

Chuong et al. recommend performing robustness evaluation to quantify changes in WET per voxel and their effect on the dose distribution [14]. This can be done by calculating the dose on two extreme breathing phases to quantify dose degradation and ensure coverage across the respiratory cycle [14,15,18].

### Quality assurance

#### Plan QA

Chang et al. and Chuong et al. recommend incorporating interplay and motion management strategies into the QA process, using a 4D-phantom [14,18].

#### Machine QA

Arjomandy et al. developed guidelines for machine QA in PT beam delivery. Their article outlines procedures for daily, weekly, and monthly QA. They suggest that trained RTTs or physics assistants may conduct these QA procedures, but the results, should be reviewed by a qualified MP [20].

### Setup and verification

Daily online imaging is recommended, preferably using 3D volumetric imaging to detect anatomical changes that affect dose distribution and assess rotation errors [13]-[15,17,19,21]. If 3D imaging is unavailable, a weekly repeat CT with dose recalculation is advised to monitor anatomical changes [15,17,19,21]. Volumetric imaging can also be used to check the consistency of the BH or gating level. When 3D imaging is not available, fiducial markers with fluoroscopic imaging can be used instead [18,19]. In prostate cancer treatment, fiducial markers can be used for image verification, and pretreatment bladder scans to ensure reproducible bladder filling [21]. Trnková et al. recommends verification before each isocenter for CSI. At a minimum, this verification should be performed using 2D kilovoltage X-ray imaging with registration based on bony anatomy. Verification imaging following a setup correction is advised in specific situations, such as machine inaccuracies or difficulties in patient positioning. Imaging dose should be considered if it exceeds a predefined threshold. Partial consensus was reached that SGPT should play a leading role in minimizing imaging dose in the future [12]. If foreign materials are in the beam path, proper alignment on these structures is essential to prevent degradation of the planned dose distribution [13].

### Evaluation during treatment course

#### Interpretation and intervention of patient changes

Posttreatment imaging in prostate cancer is recommended to ensure the margins used account for position deviations [21]. However, in CSI, consensus has been reached to perform post-fraction imaging only in certain situations, which are not further specified [12]. Acquiring regular repeat CTs with recalculation of the treatment plan is recommended to determine whether adaptive replanning is needed to maintain target coverage and to avoid overdosing OARs [13]-[16,18]. Repeat CTs should be carefully assessed for changes in anatomy and motion patterns. Changes could require further systematic monitoring and timely adjustment of the treatment plan [14]. In breast cancer treatment, clinical examination and surface imaging can identify anatomical changes [16]. Table 8 summarizes the recommended frequency of repeat CTs for various indications.

**Table 8:** Recommendations on frequency and timing of repeat CTs during treatment of various indications.

*Author*	*Population*	*Frequency repeat CTs*	*Timing first repeat CT*
Chang et al.	Thoracic	Once a week	*NR*
Chhabra et al.	Spinal tumors	Based on clinical factors	If significant changes are observed
Choi et al.	LGI	Every two weeks	Mid-treatment (minimum)
Chuong et al.	Esophagus	Every week	First two weeks
Mutter et al.	Breast	Periodic	*NR*
Zeng et al.	Mesothelioma	*NR*	First week

NR = not reported.

Acquiring repeat CT with the same BH is advised to verify consistency of diaphragm position [14]. For LGI, verification scans in the first weeks are recommended if significant rectal distention was observed during simulation, the use of an enema may be considered. In hypo fractionated treatments verification scans on day one and two are advised. [17]. For CSI, the same CT protocol as the planning CT should be used for repeat scans [12]. Choi et al. suggest using CBCT for adaptive planning, by registering it to the planning CT to evaluate and assess the need for adaptation [17].

#### Adaptation

If target coverage is compromised, or critical organs do not meet the clinical criteria, adaptation of the treatment plan is needed [13,14,16]-[19]. Chuong et al. recommends to make an adaptive plan according to the same techniques as used for the initial plan and the same patient-specific QA process should be repeated [14].

## Discussion

Our search identified guidelines relevant to the roles of RTTs working in PT. Due to varying roles and responsibilities across countries and institutions, we included articles that broadly address the roles of RTTs, as well as tasks where responsibilities lie with ROs or MPs but are implemented by RTTs. Although this review excludes photon therapy guidelines, we acknowledge their potential applicability to PT. When developing guidelines for RTTs in PT, existing photon therapy guidelines should be reviewed and adapted to the PT requirements.

This review identified 10 articles proposing guidelines related to the work of RTTs in PT. For several disease sites no guidelines appear to have been published (e.g. neurology, head-and-neck). The quantity of guidelines for each workflow step varies significantly. In literature, research often focuses on differences between photon and proton therapy, potentially resulting in certain workflow steps being highlighted more than others. For image processing, it can be assumed there are no substantial differences in procedures, explaining why only limited PT-specific guidelines are presented. On the other hand, the relatively high number of guidelines on TP can be attributed not only to the greater differences between photon and PT, but also to the involvement of MPs in TP in many countries. MPs often contribute to or lead the development of guidelines, which may account for the higher number of publications in this area. For future guideline development in TP, the paper by Korevaar et al., which proposes a method for proton plan evaluation, can serve as a basis, as it already forms part of a Dutch national consensus [22]. Furthermore, planning challenges can be organized as done by Stock et al. They have shown the level of harmonization that can be achieved by harmonizing the TP protocol for head and neck cancer [23].

Many guidelines presented here often outline what should be done but lacking detail on how to implement tasks. For example, eight articles agree on the necessity of daily image verification, but none provide detailed instructions for image assessment or responding to observed changes, which are critical in clinical practice. Protocols developed in photon therapy, for example by Buijs et al., may offer useful models for PT [24]. The lack of detail may be due to differences in equipment across centers. An example of this can be found in a paper of Bolsi et al. investigating practice patterns of image guided PT (IGPT) in Europe. They found considerable variation in IGPT techniques due to differences in equipment [6]. It is crucial to consider these factors when developing guidelines. Ideally guidelines would be tailored to specific types of equipment, but this is likely difficult to achieve. However, best practices and general guidelines could be further refined through initiatives such as exchange programs, like those organized within the TaSeRnet project. In these programs, RTTs can participate in job exchanges with centers using the same equipment, collaboratively refining guidelines to address the specific capabilities and limitations of their own systems.

Guideline quality was assessed using the AGREE II instrument. Despite involving only two reviewers, their high agreement suggests acceptable reliability. Quality scores were relatively low, mainly due to insufficient described methodologies. Therefore, it cannot be concluded that the guidelines presented here have been developed in such a way that they can be adopted without evaluating the consensus in the PT community. The article by Trnková et al. highlighted consensus areas for IGPT in CSI. However, the development of guidelines has yet to take place [12]. As a result, the AGREE II instrument was not fully applicable to this study, leading to lower scores in domains such as methodology and applicability.

Only several overlapping guidelines were identified, as the reviewed articles focused on different indications. However, the overlapping guidelines do not contradict one another and could therefore be implemented in current clinical practice. The remaining guidelines, however, require further validation before they can be adopted as best practices.

To our knowledge, only two of the articles included had one RTT as a co-author. This is particularly noteworthy given that RTTs play a significant role in both the treatment preparation and delivery. Without their involvement in guideline development, crucial insights may be overlooked, as RTTs possess specific practical knowledge and experience. For instance, no consensus was reached, and no guidelines were identified on treatment execution, which is managed exclusively by RTTs. Currently, various groups are working on identifying practices and developing guidelines, and we strongly recommend involving RTTs in this process.

In the TaSeRnet project, the outcomes of this review are used to inform a Delphi consensus study on best practices. The resulting consensus will form the basis for the development of guidelines. Based on these guidelines, educational materials will be created to promote the standardization and harmonization of practice. Additionally, it is important to note that due to rapid technological developments, the roles and responsibilities of RTTs are evolving, making it essential to develop education and guidelines that reflect these changes [25].

## Conclusion

In conclusion, although guidelines exist for several aspects of PT, the lack of detailed descriptions of the methodologies used in their development, combined with the absence of guidelines for certain workflow steps, highlights the need for further developing guidelines and best practices for RTTs. The minimal RTT involvement in developing these guidelines underscores the need for a more inclusive, multidisciplinary approach to consensus-building, ensuring that the unique expertise of RTTs is integrated into the decision-making process. Therefore, one of the key challenges ahead is establishing robust, RTT-focused guidelines, which are essential for improving the quality and consistency of PT and ultimately enhancing patient outcomes.

During the preparation of this work the author used ChatGPT in order to improve readability and language. After using this tool, the author reviewed and edited the content as needed and take full responsibility for the content of the publication.

## Declaration of competing interest

The authors declare the following financial interests/personal relationships which may be considered as potential competing interests: [This research was conducted as part of the “Towards a Sustainable RTT network” (TaSeRnet) project, which is financed by the European Union under grant agreement number 2023-1-NL01-KA220-HED-000158704. Authors who received funding through this project: EW, JJ, CX, IK, HB, MH.Hoogeman reports research grants paid to the institute from Varian, RaySearch, Accuray, Elekta, and Siemens Healthineers].

## Footnotes

1 In this article the term RTT refers to professionals involved in the preparation and delivery of proton therapy, including radiation therapists, dosimetrists, and nurses.

2 For simplicity, both guidelines and recommendations are referred to as “guidelines” in this article.
